# The New York Institute of Stomatology

**Published:** 1901-12

**Authors:** 

**Affiliations:** The New York Institute of Stomatology


### Reports of Society Meetings.

### THE NEW YORK INSTITUTE OF STOMATOLOGY.

A MEETING of the Institute was held at the office of Dr. E. H. Babcock, No. 140 Remsen Street, Brooklyn, N. Y., on Tuesday evening, June 4, the President, Dr. J. Morgan Howe, in the chair.

The minutes of the last meeting were read and approved.

*Dr. C. O. Kimball.*—I was asked a short time since by the members of the family of the late Dr. Dunning to prepare a little sketch of his life for publication, and at the request of the officers of the Institute I will read it to you. I have here a copy of a crayon portrait of Dr. Dunning, taken in his prime by one of his artist friends, and reproduced by photography within the last year. It may interest the gentlemen here to know that Dr. Dunning's grandson, one of our *confrères* in the city, is with us to-night, and has with him an old hand-glass used by Dr. Dunning. It has on the reverse side, engraved in the silver back, a list of Dr. Dunning's charges for operations when he began his work in New York nearly sixty years ago. It is a rather interesting memento which Dr. William B. Dunning has been kind enough to bring with him.

See page 808 and frontispiece.

*The President.*—We are very much obliged to Dr. Kimball for this sketch of Dr. Dunning's beautiful life, and also to Dr. William B. Dunning for showing us this article which Dr. Dunning, Sr., must have handled so many times.

We will, without waiting for other communications, proceed to the further order of business, which will be a paper by Dr. D. D. Smith, of Philadelphia.

(For Dr. Smith's paper, see page 817.)

DISCUSSION.

The following letter was received from Dr. Marshall:

"The announcement of the June meeting in your Institute of Stomatology is at hand. The special subject up for discussion,—namely, Oral Prophylaxis,—is one that interests me very greatly, and I should be exceedingly pleased if I could be present at the meeting. I feel that upon this particular line our profession must do more work than it has done if we are ever to succeed in effectually stemming the tide of the rapid decay of the teeth of the present generation. We must, however, go deeper than mere local treatment for the prevention of caries and other oral diseases. We must begin with the mothers before the children are born, and follow it up throughout the period of the growth and development of the child. Little children should begin the cleansing of their teeth as soon as they are able to hold a tooth-brush; but if the teeth are defective in development, the task of preventing disease and repairing its ravages is much greater than would be the case if the teeth were perfectly developed.

"I shall look with interest for the report of this meeting in the dental journals.

"Very truly yours,

"JOHN S. MARSHALL."

Dr. Smith presented to the Institute some cases from his private practice illustrating the results of his methods of treatment.

*Dr. Smith.*—Gentlemen, it has been suggested that I have not been sufficiently explicit in describing methods. I do not know whether I can meet your desires in this direction, as it is something which each individual must acquire for himself through practice. It is all-important to bear in mind that the tooth has not only labial and buccal surfaces, but lingual, palatal, and approximal surfaces as well, *all of which must be thoroughly treated.* I accomplish this by means of these little porte-polishers; they are a modification of the old Jack porte-polisher, and are now manufactured by J. W. Ivory, of Philadelphia. I keep eight of them with different shaped points ready for use. For the better protection of this instrument, the shaft inside the handle should be coated with white petroleum, which is a very excellent preservative for the steel, and thus very serviceable. These sticks are charged thoroughly with such pumice-stone as we ordinarily use for polishing purposes; it should not be too fine. A pumice with some grit is the best. The polishing should be done with considerable pressure, this being an important part of the operation. "How do you polish between the teeth?" is often asked. This can be done with the sticks with points thinned and shaped to suit the conditions. It cannot be done with silk or tape or with anything that I know of as well as with these same orange-wood sticks.

In pyorrhœa cases I have a remedy of my own which I call *Zhongira,* and which I have been prescribing with great satisfaction for eighteen years. The principal ingredient of this preparation is zinci chloridi. I should like to have any of the gentlemen present take one of these bottles and try it. I use it full strength for pyorrhœa and dilute about one-half for a mouth-wash.

*Dr. Allan.*—When the approximal surfaces are touching, what method do you use? Can you then polish between the teeth with a stick?

*Dr. Smith.*—I use the sticks exclusively. I find that in the progress of the treatment the sticks will go in between the teeth in a way to effect a perfect change of environment.

*Dr. Allan.*—Do you place the sticks in the hands of the patients?

*Dr. Smith.*—No. My patients use the brush only. And just here a word may be said about brushes. There are a great many badly shaped, inefficient, and unsatisfactory tooth-brushes, as there are many worthless tooth-powders. There is perhaps no better tooth-powder than that known as Caulder's dentine. There is also an excellent dentifrice sold under the name "Odontine."

*Dr. Allan.*—Do you put any pumice in your tooth-powder?

*Dr. Smith.*—No. I think pumice should be used only by the operator.

*Dr. S. E. Davenport.*—Dr. Smith has mentioned benefit coming to the gums and alveolar process by this special treatment of the teeth. I would like to ask Dr. Smith whether this benefit derived by the gums in this treatment is entirely indirect because of this treatment to the tooth-surfaces, or whether he gives any special treatment to the gums themselves and through them to the alveolar process.

*Dr. Smith.*—No, gentlemen, I do not give the gums any special treatment; the excretion from the gums collecting around the necks of the teeth is removed in this treatment, and thus they are greatly benefited. If you will allow the illustration, the finger-nails neglected and left from week to week with indifferent washing only, will show a condition not unlike gum tissue which is irritated by the perpetual presence of this foreign toxic matter. The nails must be polished to keep them in a proper condition. It is the same with the teeth. When we remove this excretion which comes from the gums, we benefit not only the gums but the alveolar process as well.

*Dr. Allan.*—About how much time did you put upon the teeth of the gentleman whom you have presented to us to-night?

*Dr. Smith.*—Before answering this last question, I should like this gentleman to make a statement, if he will, of his personal feelings in regard to his condition.

*Statement.*—I came to Dr. Smith on the 19th of March with my mouth in very bad condition, especially my gums. They had been treated for pyorrhœa alveolaris for a long time. I think I had this disease in a modified form at twenty-five. My last dentist claimed that it was constitutional in origin, and treated it accordingly. At first I thought I received some benefit, but in the end it amounted to nothing.

*Dr. Allan.*—Did he use any instrumentation?

Well, I was very much disappointed with his instrumentation; indeed, at the same time that I was going to the gentleman named I used to go to another doctor to have the tartar removed, because I thought the first doctor was not thorough enough. My mouth has been perfectly comfortable since I have been under treatment by Dr. Smith.

*Dr. Kimball.*—At what intervals has this mouth been treated?

*Dr. Smith.*—The treatment at first consisted in thorough removal of all foreign matter on and about the teeth and roots, the mouth being frequently rinsed with phénol sodique, full strength. For two weeks I saw the patient three times a week, each time going over *all* the surfaces of the teeth and down into the pus-pockets with the sticks and pumice. Bleeding, which was quite free in the beginning, soon ceased entirely, and the gums began to take on a normal condition. Each time after polishing out with the stick and pumice the pockets were touched with deliquesced zinci chloridi *applied with a stick.* At the end of two weeks the treatment was changed to twice a week for four weeks, when it was reduced to once a week, and this has been continued to the present time, interrupted only by a very few necessary absences from the city. When he first presented, a thick, gummy, mucoid secretion would quickly recement itself on the teeth after its removal. This condition is now apparently cured. This *frequency* of cleansing is one of the points upon which I have been criticised, some maintaining that it will result disastrously to the gums. I assure you, gentlemen, that it will do no harm. The excretion from these diseased gums must be removed, and that frequently.

*Dr. Kimball.*—Do you use any obtundent in extremely sensitive cases?

*Dr. Smith.*—I do not use anything at all except phénol sodique. I have never found a case I could not treat without special pain. Patients find great comfort and relief in using the "Zhongira," to which allusion has been made.

*Dr. F. Milton Smith.*—Do you employ the engine?

*Dr. Smith.*—Most decidedly, no.

*Dr. Eames.*—Do you polish way down to the end of the pocket, or do you expect the pocket to close?

*Dr. Smith.*—I polish the pockets gently but thoroughly. It is a question if the gum structure will attach itself to the tooth, but if it does not, there will be a reshaping of the process and gums so as to obliterate the pocket.

*Dr. F. Milton Smith.*—May I ask your reason for never using the engine?

*Dr. Smith.*—Because the desired results cannot be obtained with it.

*Dr. F. Milton Smith.*—As I understand this treatment it consists of thoroughly cleansing the teeth, followed by what Dr. Smith calls massage, which consists of rubbing the surfaces of the teeth. If this is the case, I cannot see why it cannot be accomplished in a time-saving way by the use of the engine.

*Dr. Smith.*—It is a mistake to attribute the use of the term massage to me in this connection. There is no saving of time in using the engine for this treatment, and the beneficial effect of stimulation to the tooth is lost. Were not this the case, it is an absolute impossibility to get any wheels run by the engine to do the work that is done on the labial, buccal, lingual, and palatal surfaces, as well as between the teeth, by these properly shaped sticks charged with pumice and intelligently and skilfully handled in these little porte-polishers. If the position of the teeth and their surfaces permitted the use of *wheels* for this purpose,—which they do not,—the resistance interposed by the involuntary muscular contraction, in which the tongue, lips, buccinator, and the floor of the mouth all join, would surely defeat that thoroughness in the operation which contemplates complete change of environment, and thorough polishing of the teeth.

*Dr. A. H. Brockway.*—I have greatly enjoyed the talk of Dr. Smith, and have also read with profit his articles in the journals on the same subject. I think he is on the right track, and I am an easy convert to his idea, for I have long believed in the efficacy of frequent polishing of the teeth. But it will be observed that Dr. Smith goes farther than simply cleaning the teeth; his idea is to produce by his manipulations a stimulating effect, as it were, to the vitality of the tooth, thus better enabling it to resist pathogenic influences. This seems plausible, and if it be so it is indeed very important.

*Dr. Kimball.*—Mr. President and gentlemen, it has been a very pleasant thing to me personally to hear this talk from Dr. Smith, and I think it has been an exceedingly instructive one to us. My mind has gone back very naturally, under the circumstances of the evening, to my preceptor, Dr. Dunning, whose portrait is before you; again and again his instructions to me have come to mind: the same care in using the stick; the same thorough system of cleansing the teeth frequently—though not so frequently as Dr. Smith has done—with orange-wood sticks held in the porte-polishers and pumice-stone, producing healthy reaction by diligent rubbing, with the result that I have been surprised in my own practice to find how few cases of pyorrhœa alveolaris I have seen. It is but a few years since I remarked to a friend that I had never seen a case of pyorrhœa alveolaris in my practice or in that which I had inherited from Dr. Dunning, because of this system of cleansing teeth which he taught and which we are endeavoring to carry out.

Dr. Smith goes farther in this, that the same care has been carried on at more frequent intervals. As I reason carefully upon Dr. Smith's statements, I think he has seen the wisdom of following this cleansing at sufficiently short intervals to prevent the reformation of this deposit on the teeth, and that it is for this special point that we have to thank him; not only the removal of the deposit, but following it up with sufficient frequency to bring about a correction in the general condition of the mouth. As regards the use of floss-silk for polishing between the teeth, perhaps if he had tried using floss-silk folded three or four times and thoroughly waxed with yellow beeswax he would find that then, with powdered pumice-stone and water rubbed into the waxed silk, he would be able to polish between the teeth very thoroughly. In this manner you can polish down under the folds of the gum. I fully agree with what Dr. Smith says about the use of the hands. My son is now a student. I shall not allow him to use the engine until he can work thoroughly well by hand. I do the same with the young men in my office. I do certain parts of this work with power-polishers, but I do it with the experience of years in using pumice-stone with sticks held in the hand. I find that after I am through with the power-polishers I am very apt to get my stick and go around in the out-of-way places, using power for the places that can be reached easily and the stick in the difficult places.

And now I wish to offer, on behalf of the Executive Committee, a most hearty and cordial vote of thanks to Dr. Smith not only for his coming here to-night, but for the great pains he has taken to prepare for us. I move you, Mr. President, that a cordial vote of thanks be extended to Dr. Smith for his excellent paper; and also that we tender our most sincere thanks to his patients for coming here and showing us this beautiful work.

*Dr. Smith.*—I thank you very much, gentlemen, for so patiently listening to what I have been permitted to say here this evening, and I sincerely hope the future will disclose that it has not been altogether in vain.

Adjourned.

FRED. L. BOGUE, M.D., D.D.S.,
*Editor The New York Institute of Stomatology.*

